# Supplementary figures and images for: Inferred expression regulator activities suggest genes mediating cardiometabolic genetic signals
Source: PLoS Comput Biol. 2021 Nov 18;17(11):e1009563. doi: 10.1371/journal.pcbi.1009563 (PMC8639061; doi:10.1371/journal.pcbi.1009563)

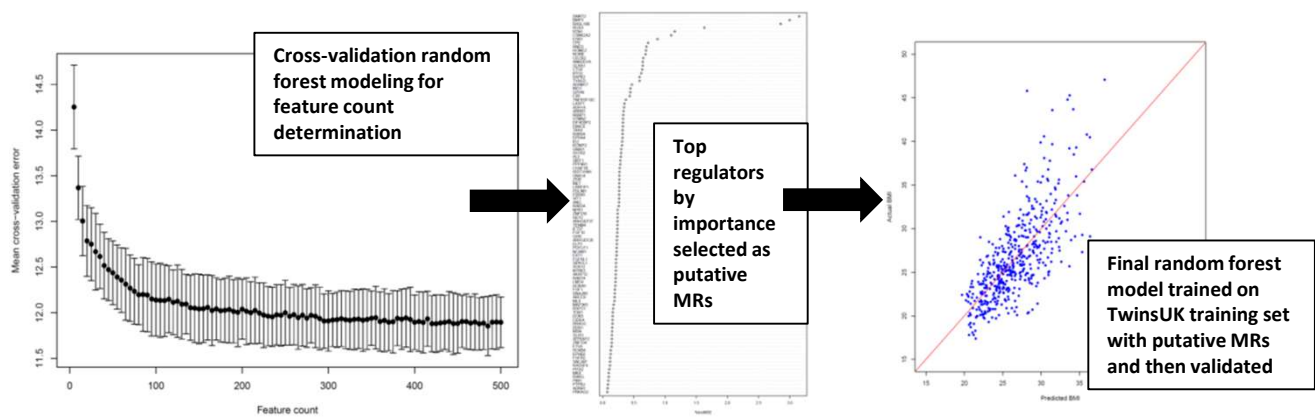

**S1 Fig**

Supplement: S1 Fig — First, test cross-validation error for random forest models with increasing numbers of regulators to determine a parsimonious number of regulators sufficient to minimize prediction error. Next, identify the most important regulators as measured by the percent increase in the mean square error (MSE) upon permutation of the regulator in all trees of the forest. Finally, train the final random forest model with the determined number of top regulators by importance and test the model in the test set and validation set. (PDF) [file pcbi.1009563.s001.pdf]

**A**

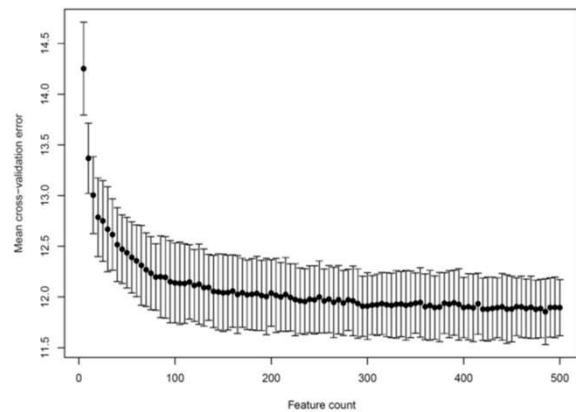

**B**

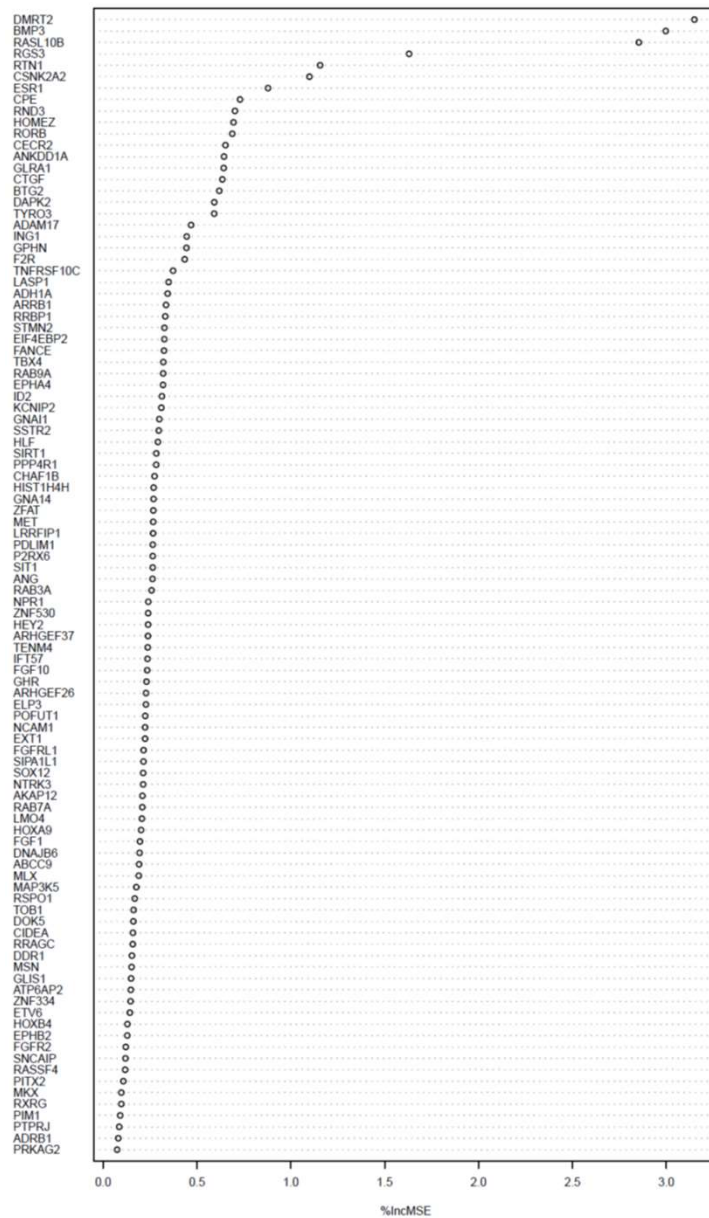

S2 Fig

Supplement: S2 Fig — (A) Cross-validated prediction performance of random forest regression models with the number of predictors sequentially reduced by five. Models were trained to predict BMI from regulator activities 12 times, each with a unique seed. The plot compares the number of predictors included in the model versus the mean cross-validation error and error bars indicate the standard deviation of the 12 analyses. (B) Rank order for the top 100 regulators by importance to BMI prediction by the final random forest model. The importance is measured as the percent increase in the mean squared error (MSE) upon permutation of the regulator across all trees of the random forest. (PDF) [file pcbi.1009563.s002.pdf]

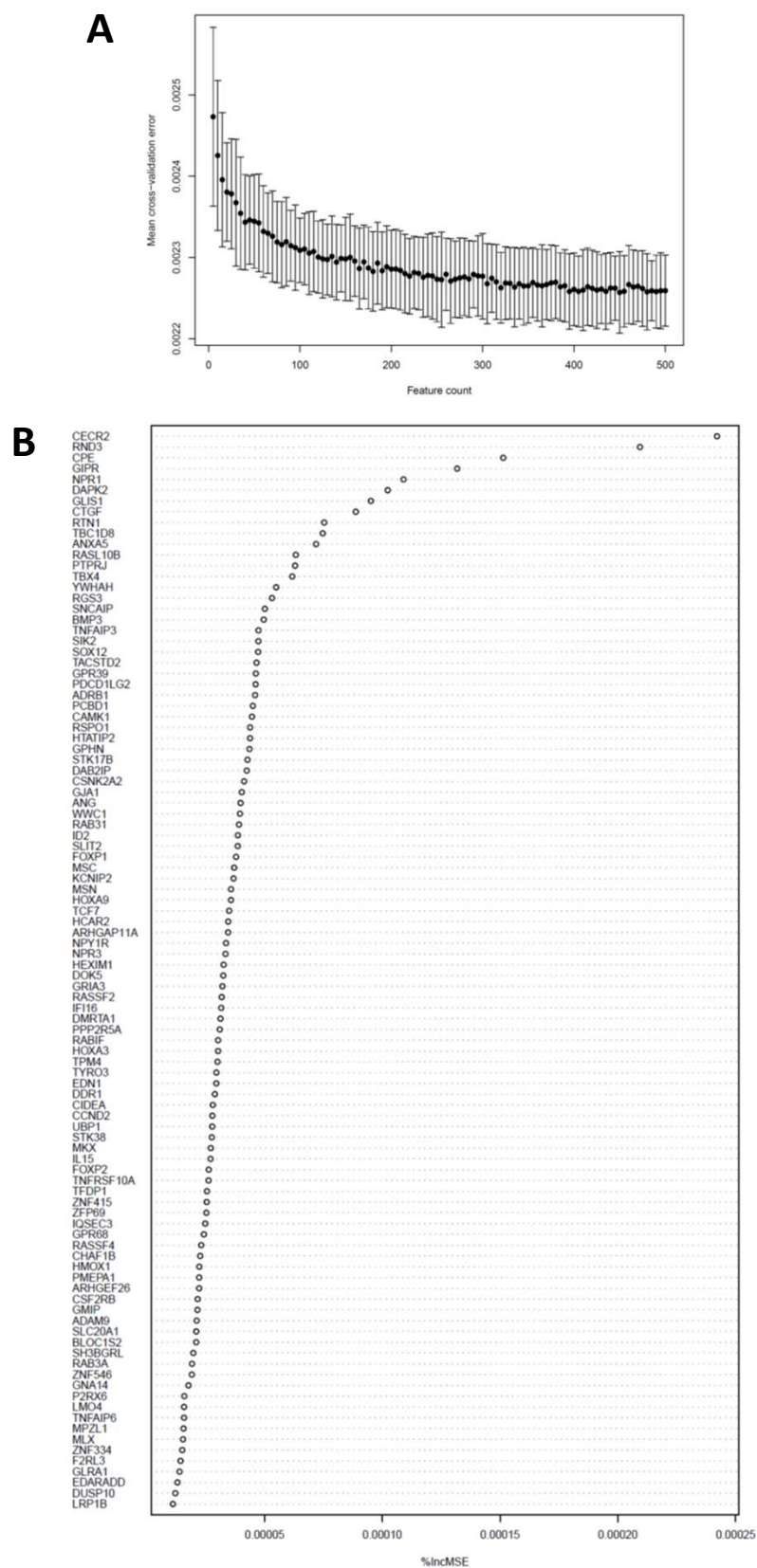

S3 Fig

Supplement: S3 Fig — (A) Cross-validated prediction performance of random forest regression models with the number of predictors sequentially reduced by five. Models were trained to predict WHR from regulator activities 12 times, each with a unique seed. The plot compares the number of predictors included in the model versus the mean cross-validation error and error bars indicate the standard deviation of the 12 analyses. (B) Rank order for the top 100 regulators by importance to WHR prediction by the final random forest model. The importance is measured as the percent increase in the mean squared error (MSE) upon permutation of the regulator across all trees of the random forest. (PDF) [file pcbi.1009563.s003.pdf]

**A**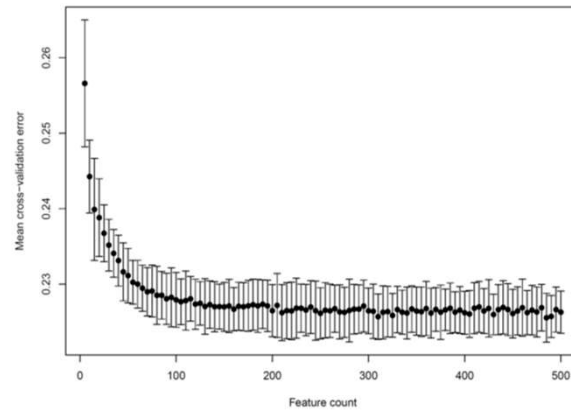**B**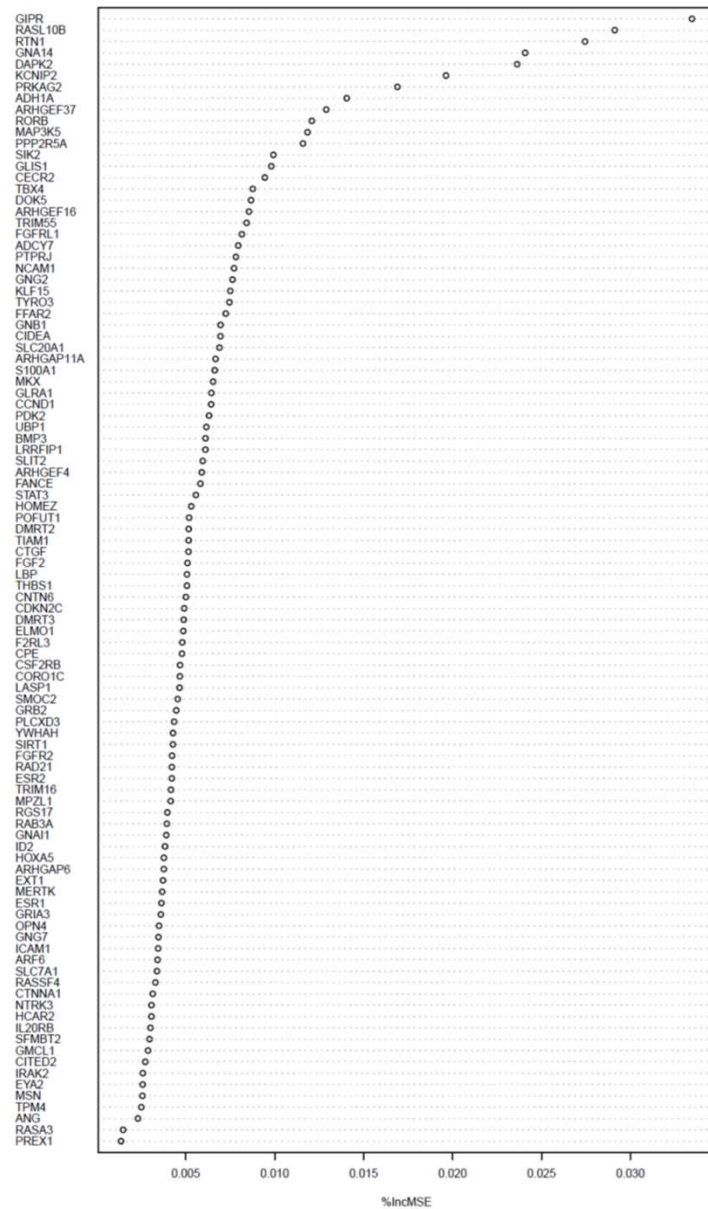

S4 Fig

Supplement: S4 Fig — (A) Cross-validated prediction performance of random forest regression models with the number of predictors sequentially reduced by five. Models were trained to predict ln(HOMA-IR) from regulator activities 12 times, each with a unique seed. The plot compares the number of predictors included in the model versus the mean cross-validation error and error bars indicate the standard deviation of the 12 analyses. (B) Rank order for the top 100 regulators by importance to ln(HOMA-IR) prediction by the final random forest model. The importance is measured as the percent increase in the mean squared error (MSE) upon permutation of the regulator across all trees of the random forest. (PDF) [file pcbi.1009563.s004.pdf]

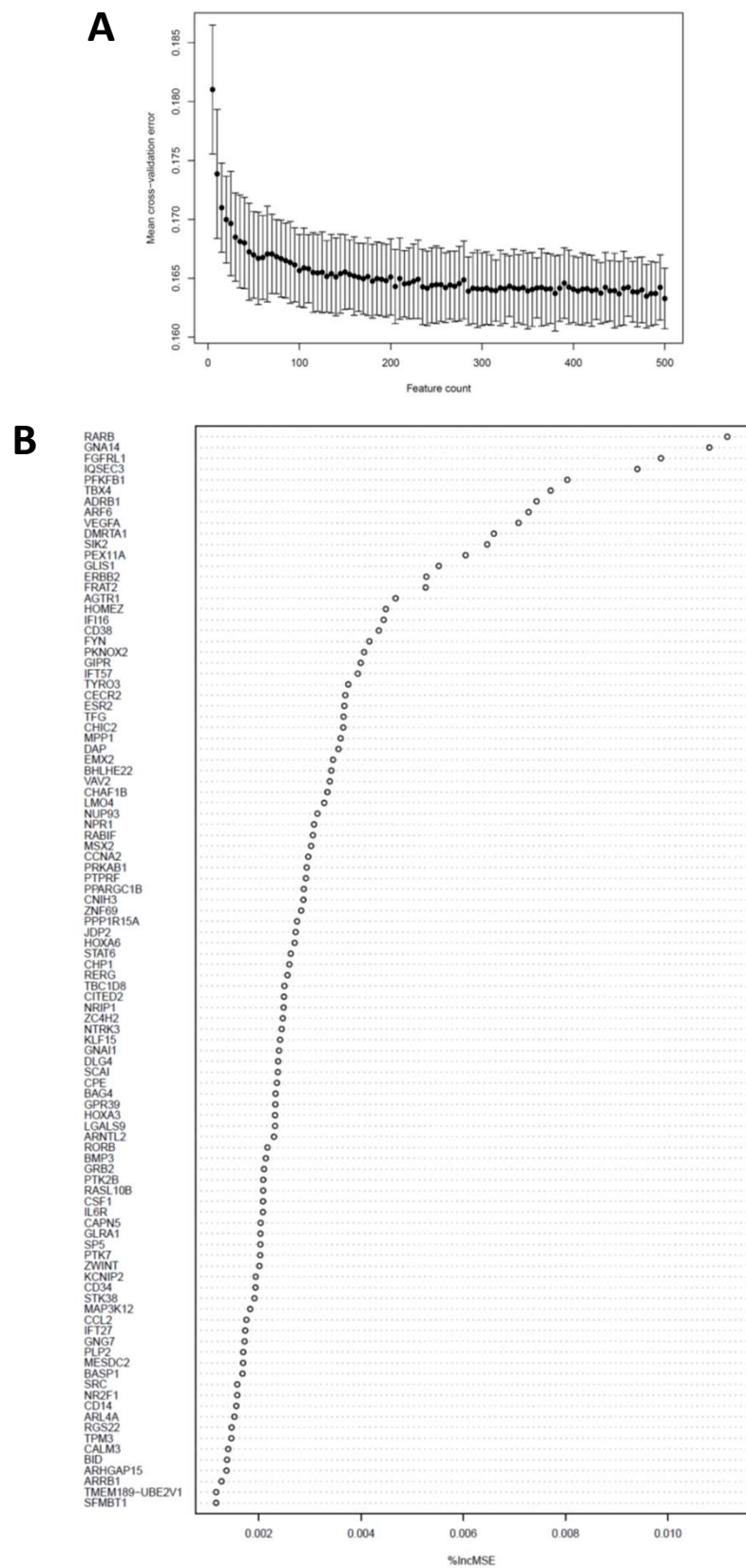

S5 Fig

Supplement: S5 Fig — (A) Cross-validated prediction performance of random forest regression models with the number of predictors sequentially reduced by five. Models were trained to predict HDL from regulator activities 12 times, each with a unique seed. The plot compares the number of predictors included in the model versus the mean cross-validation error and error bars indicate the standard deviation of the 12 analyses. (B) Rank order for the top 100 regulators by importance to HDL prediction by the final random forest model. The importance is measured as the percent increase in the mean squared error (MSE) upon permutation of the regulator across all trees of the random forest. (PDF) [file pcbi.1009563.s005.pdf]

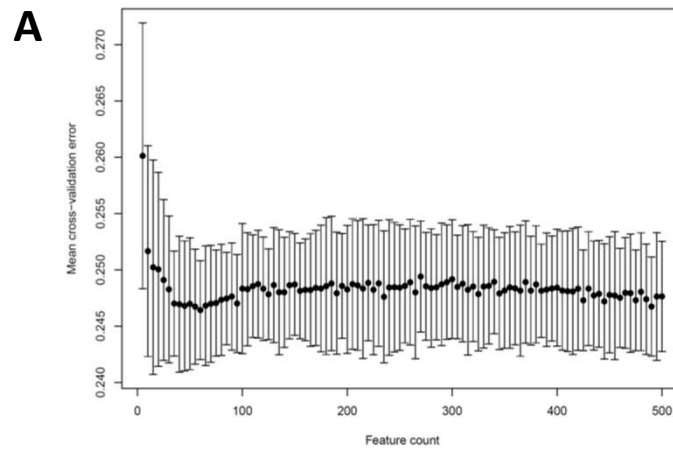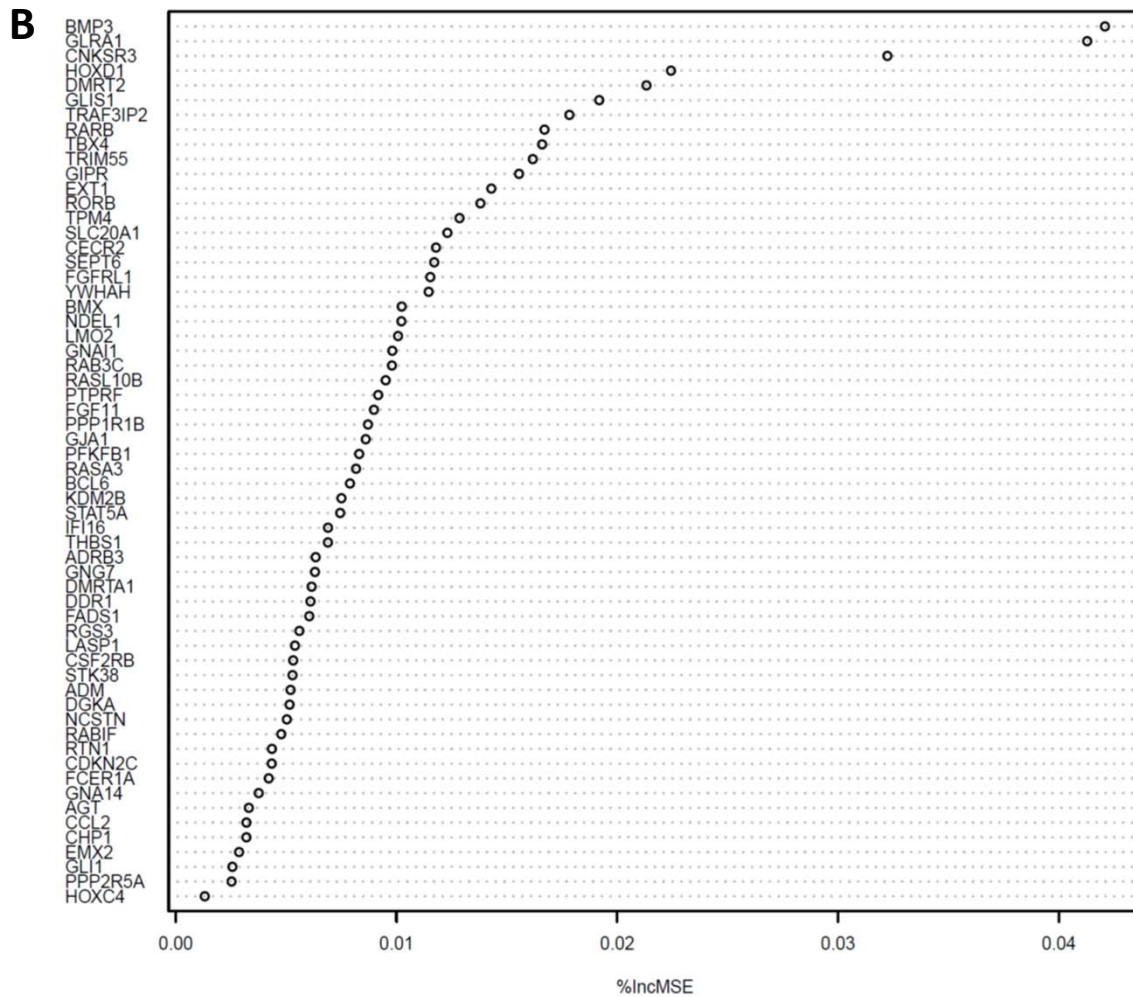

S6 Fig

Supplement: S6 Fig — (A) Cross-validated prediction performance of random forest regression models with the number of predictors sequentially reduced by five. Models were trained to predict triglycerides from regulator activities 12 times, each with a unique seed. The plot compares the number of predictors included in the model versus the mean crossvalidation error and error bars indicate the standard deviation of the 12 analyses. (B) Rank order for the top 100 regulators by importance to triglycerides prediction by the final random forest model. The importance is measured as the percent increase in the mean squared error (MSE) upon permutation of the regulator across all trees of the random forest. (PDF) [file pcbi.1009563.s006.pdf]

A

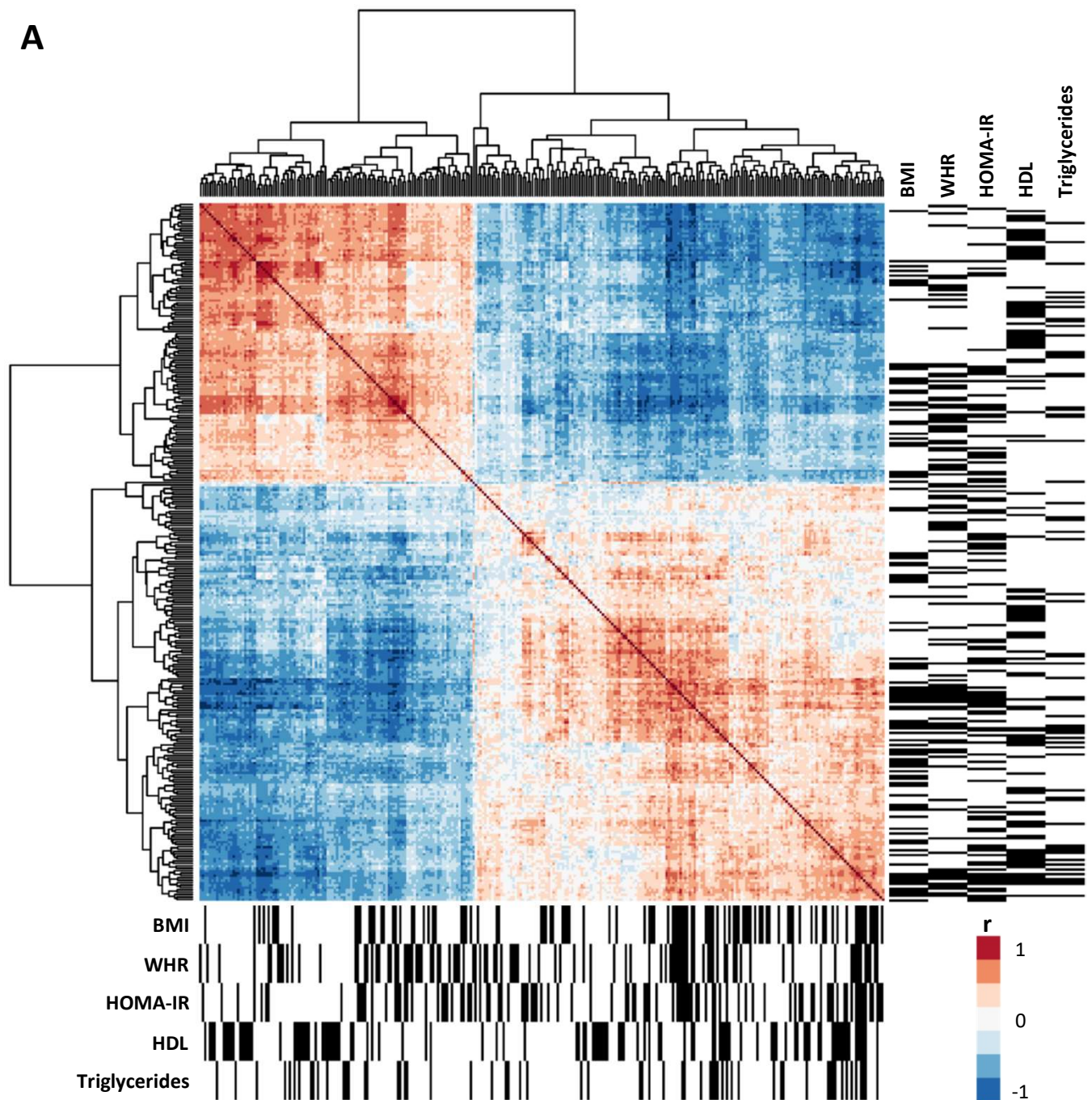

S7A Fig

**S7B Fig**

C

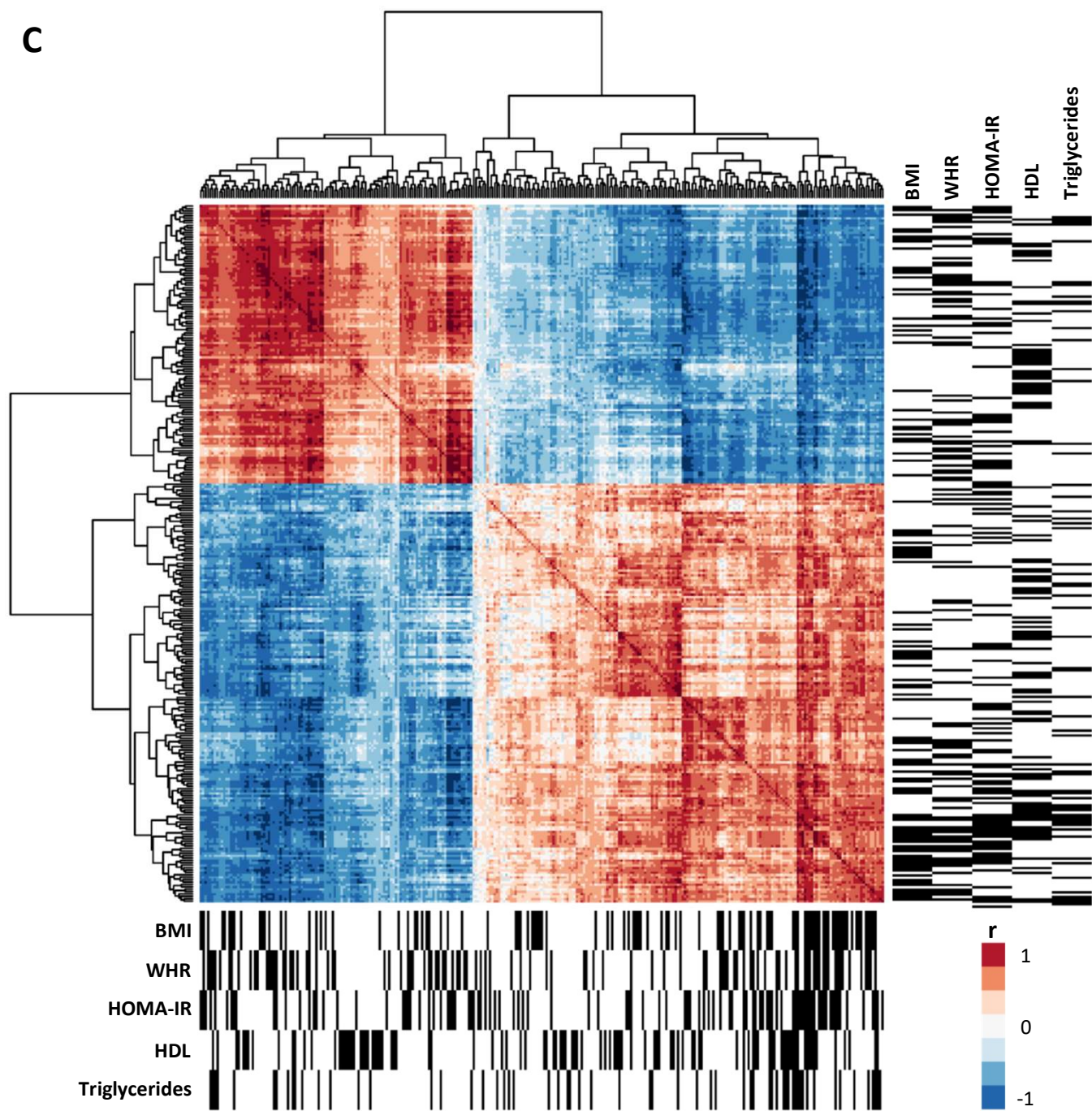

S7C Fig

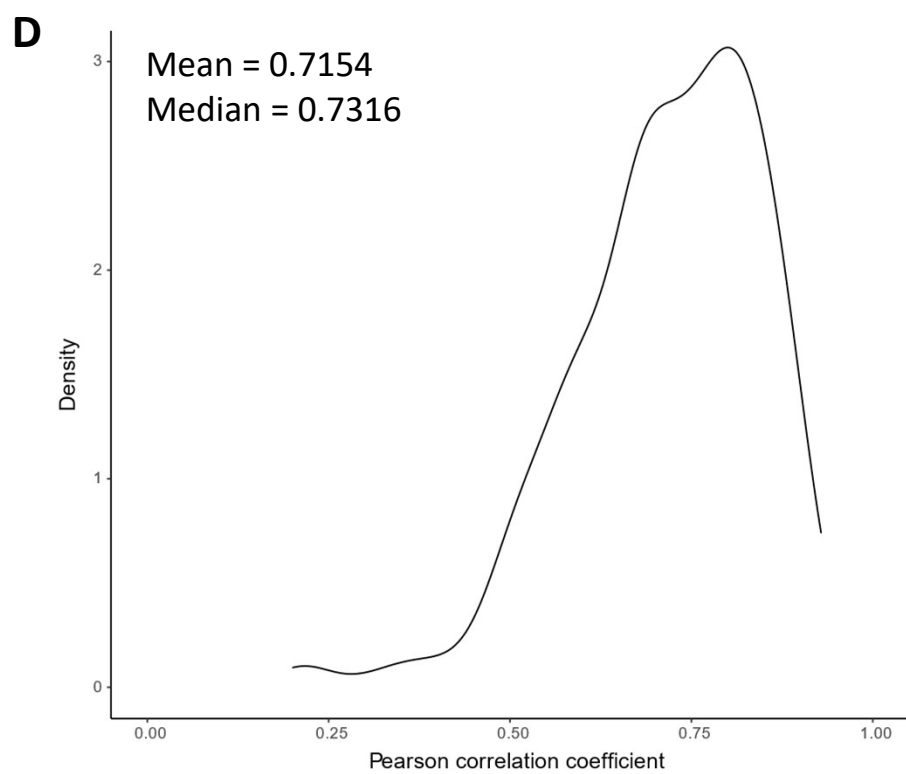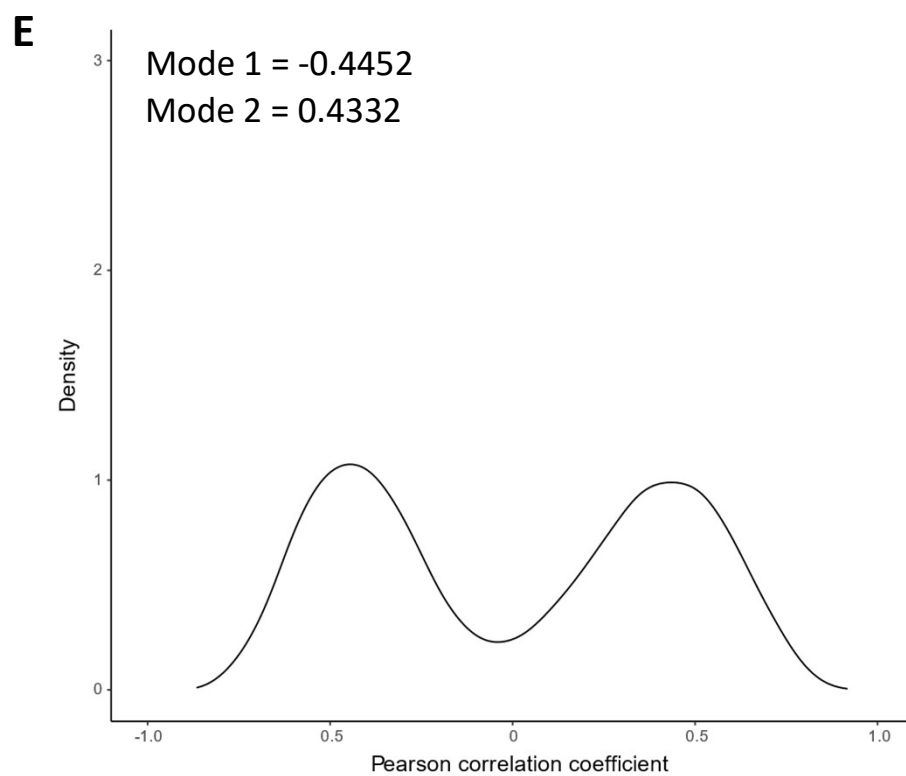

**S7D and S7E Fig**

**F**

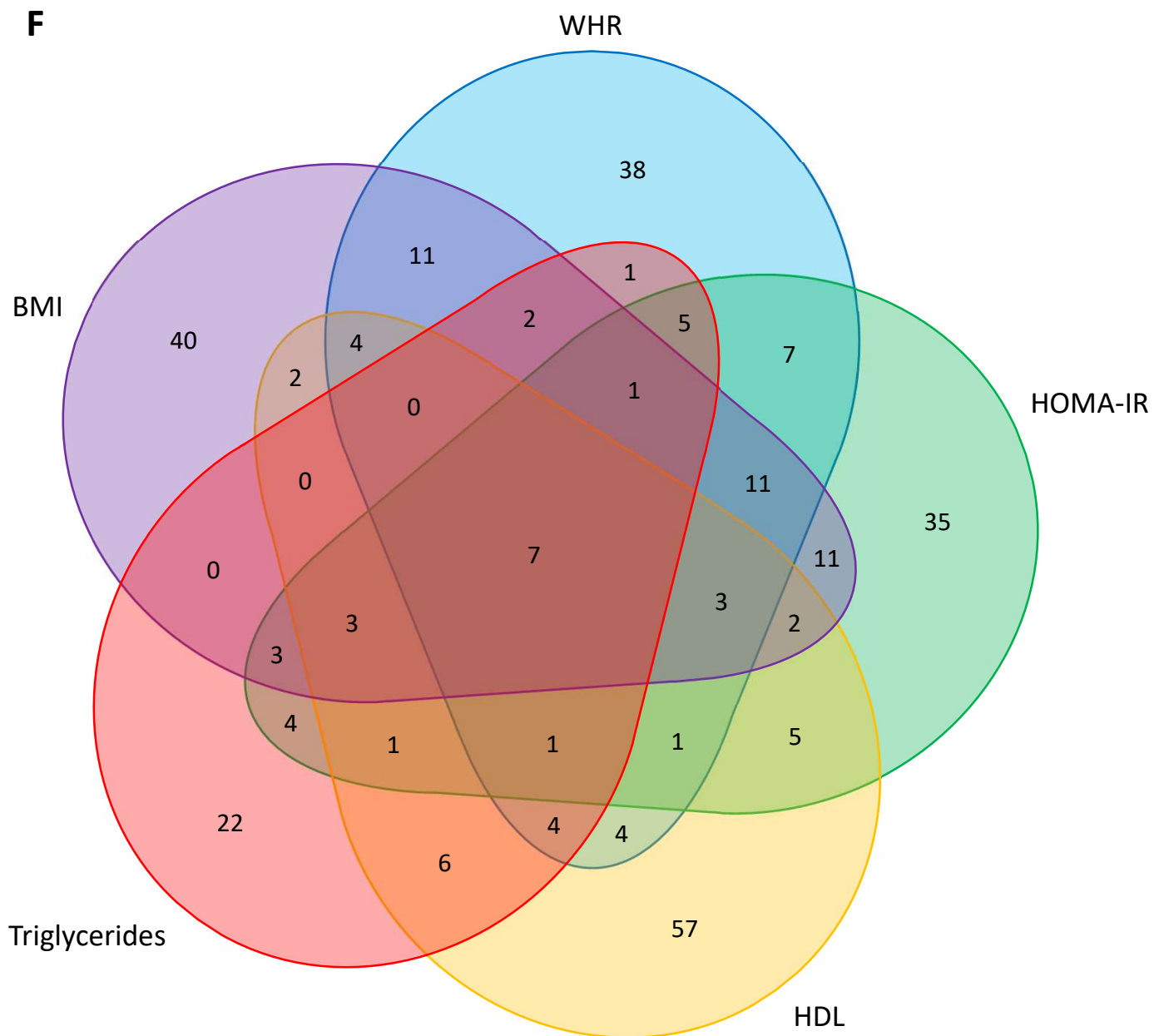

S7F Fig

Supplement: S7 Fig — (A) Clustered heatmap of Pearson correlations between the expression profiles for putative phenotypic master regulators in the TwinsUK adipose samples. The black bars to the right and bottom of the heatmap indicate for which phenotype each regulator was identified as a candidate master regulator. (B) Same as A, but between the activity profiles for putative phenotypic master regulators in the TwinsUK adipose samples. (C) Same as A, but between the expression profiles (for regulators indicated by row)and activity profiles (for regulators indicated by column) of putative phenotypic master regulators in the TwinsUK adipose samples. Note that the correlation coefficients for the heatmap in S7C Fig are not symmetrical about the diagonal since correlation between the expression profile of a row regulator and the activity profile of a column regulator is not the same as the converse, except along the diagonal (where the row and column regulators are identical). Consequently, though the rows and columns are in the same order, the clustering is based solely on columns (i.e. the vectors of correlation coefficients for the column regulators’ activity profiles versus the expression profiles for the row regulators). (D) Density plot of Pearson correlation between matched expression and activity for master regulators. (E) Density plot of Pearson correlation between unmatched expression and activity for master regulators. (F) Venn diagram demonstrating the number of master regulators that are unique or in common among the phenotypes analyzed. (PDF) [file pcbi.1009563.s007.pdf]

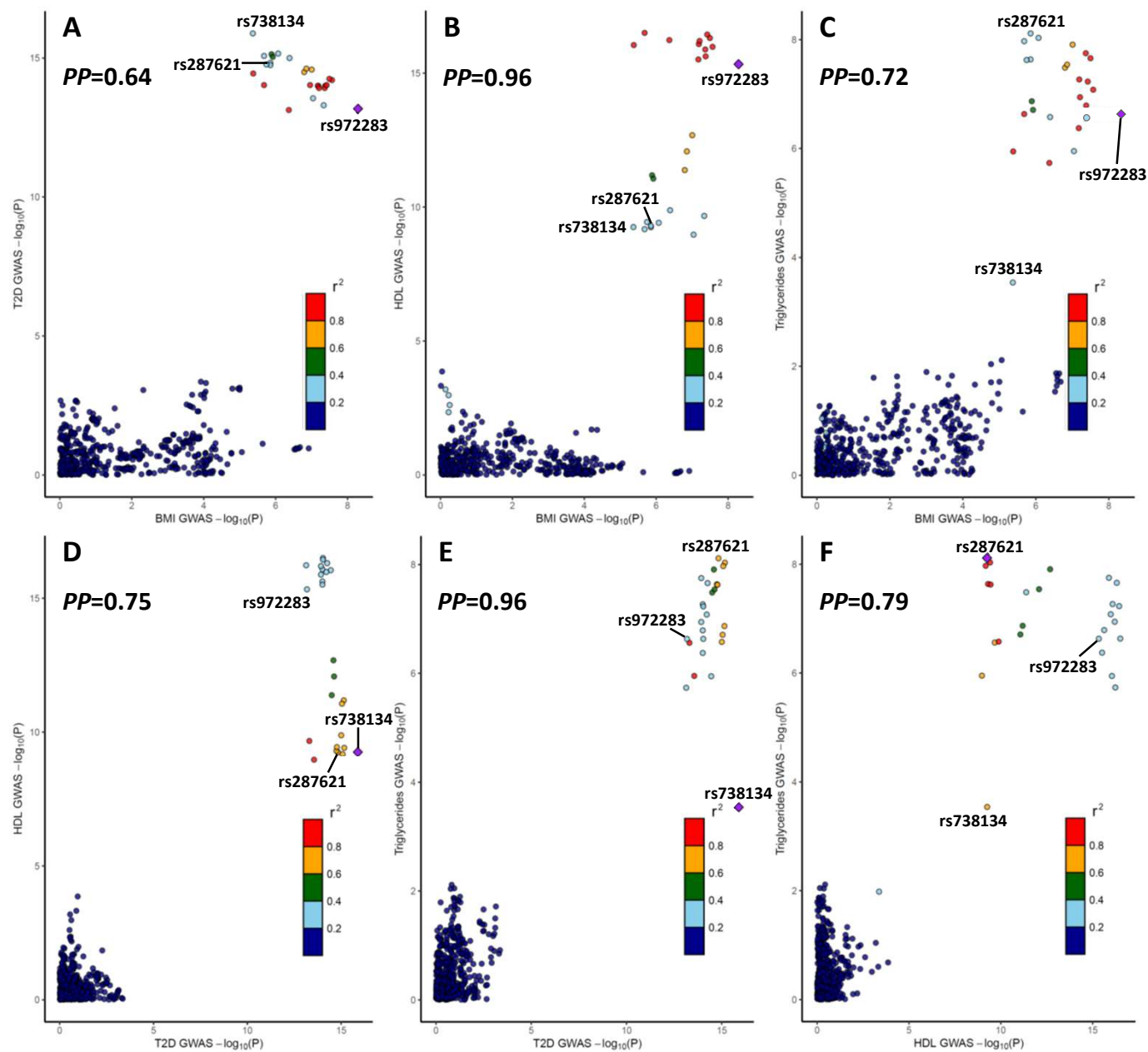

S8 Fig

Supplement: S8 Fig — (A) LocusCompare plot of chr7q32.2 locus variants’ -log10(P) from BMI GWAS versus T2D (BMI-adjusted) GWAS. Points are colored according to the variant’s r2 with the reference variant, which is marked by the purple diamond. Posterior probability (PP) of a single, shared functional variant was calculated with HyPrColoc. (B) Same as A, but for BMI GWAS versus HDL GWAS. (C) Same as A, but for BMI GWAS versus Triglycerides GWAS. (D) Same as A, but for T2D (BMI-adjusted) GWAS versus HDL GWAS. (E) Same as A, but for T2D (BMI-adjusted) GWAS versus Triglycerides GWAS. (F) Same as A, but for HDL GWAS versus Triglycerides GWAS. (PDF) [file pcbi.1009563.s008.pdf]

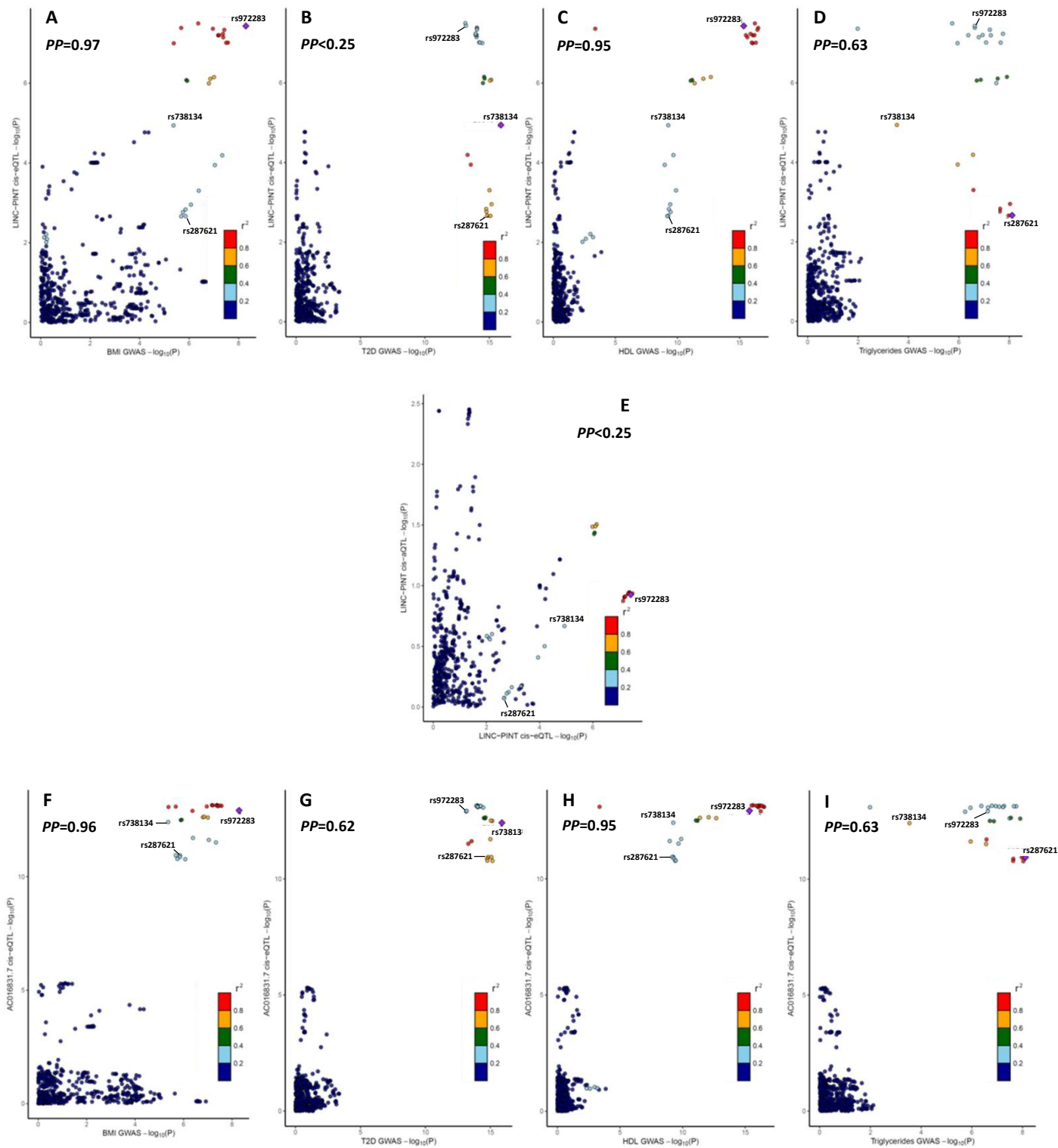

S9 Fig

Supplement: S9 Fig — (A) LocusCompare plot of chr7q32.2 locus variants’ -log10(P) from BMI GWAS versus LINC-PINT cis-eQTL. Points are colored according to the variant’s r2 with the reference variant, which is marked by the purple diamond. Posterior probability (PP) of a single, shared functional variant was calculated with HyPrColoc. (B) Same as A, but for T2D (BMIadjusted) GWAS versus LINC-PINT cis-eQTL. (C) Same as A, but for HDL GWAS versus LINC-PINT cis-eQTL. (D) Same as A, but for Triglycerides GWAS versus LINC-PINT cis-eQTL. (E) Same as A, but for LINC-PINT cis-eQTL versus LINC-PINT cis-aQTL. (F) Same as A, but for BMI GWAS versus AC016831.7 cis-eQTL. (G) Same as A, but for T2D (BMI-adjusted) GWAS versus AC016831.7 cis-eQTL. (H) Same as A, but for HDL GWAS versus AC016831.7 cis-eQTL. (I) Same as A, but for Triglycerides GWAS versus AC016831.7 cis-eQTL. (PDF) [file pcbi.1009563.s009.pdf]

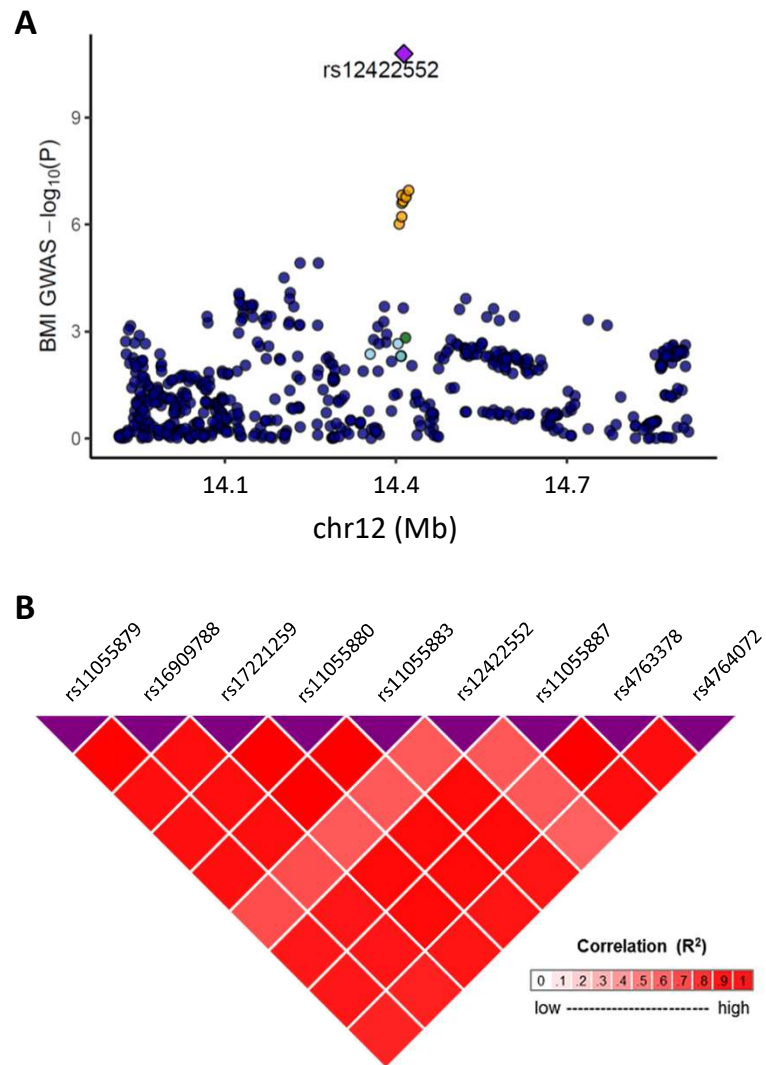

S10 Fig

Supplement: S10 Fig — (A) Plot of genomic position versus BMI GWAS -log10(P) for variants at the chr12p13.1 locus. (B) LD plot between top nine BMI GWAS variants at the chr12p13.1 locus shaded by pairwise r2. (PDF) [file pcbi.1009563.s010.pdf]
