# Supplementary material for: Inferred expression regulator activities suggest genes mediating cardiometabolic genetic signals
Source: PLoS Comput Biol. 2021 Nov 18;17(11):e1009563. doi: 10.1371/journal.pcbi.1009563 (PMC8639061; doi:10.1371/journal.pcbi.1009563)
Supplement: Attachment — Submitted filename: comments.docx [file pcbi.1009563.s033.docx]

Hoskins et.al., proposed an “activity QTL(aQTL) analysis” and hypothesized that genetic variants regulating disease-associated master regulators(MR) could underlie the GWAS signals for metabolic traits. The authors constructed gene co-expression network in forms of regulator-> targets connections at subcutaneous adipose, a tissue highly relevant to metabolic traits. They then inferred activities of regulators from the network. They next applied random forest regression to identify top regulators(MR) for each metabolic trait. With those information they performed analyses to identify cis/trans eQTLs/aQTLs that potentially regulate the expression or the activity of MRs in subcutaneous adipose and to test whether and how those QTLs colocalize with GWAS hits of metabolic traits. They found whilst in cis, regulatory variants that colocalize with GWAS hits seem to have a stronger effect for expression of MRs than for activity of MRs, in trans those variants tend to have a stronger effect for activity than for expression of MRs. They also provided examples where MR trans-QTLs(eQTLs or aQTLs) provided intriguing clues of the functional relevance of GWAS loci when cis-QTLs provided limited information. The authors concluded that these analyses can be broadly applied given the availability of data(expression and GWAS data) for many complex traits, and that expression/activity-type analyses of MRs add additionally valuable findings to classical global eQTL analyses.

Overall, I think this study proposed advanced and interesting concepts (aQTLs, MRs) and provided promising application (colocalization with GWAS signals). The analyses were carefully designed and the results are appropriately discussed. I have some questions which would like the authors to consider:

1. The author claimed that overall trans-aQTLs tend to be more significant/stronger than their respectively eQTLs. Was this conclusion drawn from raw p values from QTL analysis, or has the effect size been compared(if both activity and expression have been normalized onto comparable scale)? According to the paper, in its nature the activity of a MR reflected expression input from its target gene members within the regulon. Would it be possible that a strong tran-aQTL signal of a MR simply tracked a strong cis-eQTL signal of one of the target genes in the regulon of that MR?
2. A relevant and important question is that which variants, those interrupting/regulating MR or those interrupting/regulating MRs’ target genes in the regulon contribute more to the genetic susceptibility of complex traits? There has been debate in the field about the omnigenetic model(or the core-gene model) <https://www.ncbi.nlm.nih.gov/pmc/articles/PMC5536862/> and the polygenicity model <https://pubmed.ncbi.nlm.nih.gov/29906445/> for genetic architecture of complex traits. What the authors explored here are essentially in line with the ‘omnigenetic model’ by showing variants regulating core-genes(master regulators) with potentially larger effect can underlie some GWAS signals. What the author haven’t explored was that whether variants in target genes of the MRs also contributed to GWAS signals (polygenicity model), and how does that compare to the contribution from MR QTLs?. In figure s7, the author observed extensive correlation between MRs, both in terms of activity and expression. This suggested relatively limited independent pathways/room to be interrupted at core-genes(MRs) level and implicate peripheral genes(target genes of MRs) can potentially be better candidates for GWAS signals.

My other minor comments/questions are:

1. L150: whether the gender differences are responsible for the performance differences can be directly tested by only including females in test dataset. HDL and Triglycerides are potentially more fluctuated than other traits though.
2. In some places the authors used ‘overlapped’ in other places they used ‘colocalized’. Are they interchangeable? Please be consistent.
3. The snp id is different in L214, legends of Figure 2 and the figure 2 itself. Why among the two independent SNPs, only one was considered?
