# Supplementary material for: Inferred expression regulator activities suggest genes mediating cardiometabolic genetic signals
Source: PLoS Comput Biol. 2021 Nov 18;17(11):e1009563. doi: 10.1371/journal.pcbi.1009563 (PMC8639061; doi:10.1371/journal.pcbi.1009563)
Supplement: Attachment — Submitted filename: Responses_to_PLOS_Comp_Biol_reviewers_9-14-2021.docx [file pcbi.1009563.s034.docx]

First, we would like to sincerely thank all four reviewers for their thorough considerations and constructive feedback. They have certainly contributed to the improvement of this manuscript.

**Reviewer #1**: Hoskins et al. leveraged an adipose-specific gene regulatory network to infer expression regulator activities and phenotypic master regulators (MRs), which were used to detect activity QTLs (aQTLs) at cardiometabolic trait GWAS loci and reported several GWAS loci colocalized with MR trans- eQTLs/aQTLs in the absence of colocalized cis-QTLs. The transformation of gene expression into activity by integrating information including gene-gene interactions may be informative for genetic researchers and provide insights for downstream analysis of GWAS loci. But some of the results in this study seem to be biased, there are some major comments as below.

1. Regulator activities were inferred from the expression data and the adipose co-expression network which was constructed using TwinsUK RNA-seq data of female samples. As far as I am concerned, estrogen has a protective effect on cardiometabolic phenotypes, which would introduce more confounding factors in this study if the regulator activities of regulatory genes were inferred solely from female data. In the “Inference of gene regulator activities from transcriptomic data” part, would it be better if the TwinsUK dataset and METSIM dataset were merged together to infer regulator activity with adjustment for sex or simply use METSIM expression array dataset which was all male samples as training set and TwinsUK as test set?

We appreciate the reviewers concern and have consequently added statements to the Results and Discussion drawing attention to the fact that these aQTL results have not been validated in a male or mixed-gender eQTL dataset. However, we do not feel this gender bias affects the main conclusions of this study for the following reasons.

The reviewers concern that an all-female adipose expression dataset will yield an ARACNe co-expression network that may differ from that of an all-male adipose expression dataset are understandable. However, when using a random forest model trained from the all-female activity profiles of the TwinsUK study to predict cardiometabolic phenotypes in the all-male METSIM dataset using activities inferred with an all-female adipose ARACNe network, the predictions were still significantly correlated with the actual phenotype measures. One reason for this success despite using a gender mismatched ARACNe network is likely the robustness of activity inferences by VIPER. Alvarez et al. demonstrated that VIPER activity inferences remained robust even when up to 60% of regulon interactions were randomized. Therefore, unless the adipose gene regulatory interactions differ by more than 60% between genders, the activity inferences are likely robust. Even so, we do not deny that MR random forest models trained in an all-male dataset would likely outperform our all-female MR random forest models when applied to an all-male dataset for phenotype prediction from activity profiles. But, apart from testing the general applicability of our phenotypic MR random forest models in an independent dataset (which did well despite the gender mismatch), the all-female adipose ARACNe co-expression network was being applied to an all-female expression dataset to infer regulator activities and perform aQTL analyses. Furthermore, attempting to infer a gender neutral adipose ARACNe network from the TwinsUK and METSIM expression datasets together would be fraught with technical challenges due largely to the differences in transcriptomic platforms (i.e., RNA-seq vs. microarray, respectively), and would probably not improve the activity inferences in the all-female TwinsUK dataset.

So, it seems that the important question is not how an all-female adipose co-expression network might bias the activities used for our aQTL analyses (which they aren’t since they are gender matched and activity inferences are robust). Rather it is how this gender bias affects the conclusions of this study. The main question asked was whether aQTLs in general and trans-QTLs focused on candidate MRs might provide any additional benefit beyond traditional eQTL studies to generating hypotheses about the genes or regulatory networks mediating the effects of GWAS signals. We concluded that aQTLs can provide additional information that aid such hypothesis generation, particular in the MR trans-QTL analyses. The cases highlighted in the manuscript served merely as examples of the sorts of hypotheses that may be generated through such a study, but they were only put forward as reasonable hypotheses that call for additional functional testing.

2. In trans-eQTL and aQTL analysis of GWAS signals, the multiple testing burden of the analysis was reduced by restricting the target genes to MRs of matching or relevant phenotypes. It is reasonable for the analysis for trans-aQTL while not appropriate for trans-eQTL because the MRs were inferred from the activities but not the expression of regulatory genes. Trans-aQTLs tend to outperform their respective eQTLs because of the biased MRs selection.

We appreciate the reviewer’s concern about the potential introduction of bias into our analysis by using MRs inferred from activities rather than expression. Such bias would certainly undermine certain conclusions of our study. First, let us note that the MR inferences are entirely agnostic regarding GWAS or any other genetic information, and as such, the metric (expression vs. activity) used in the MR inference should not bias which metric has better genetic correlations for any particular gene-locus pair. Therefore, the concern voiced here is about systematic bias being introduced by using regulators selected for having better correlation between the GWAS trait and the regulators’ activity than with their expression values. To address this, we now also report the effects of using expression-based putative MRs for *trans*-QTL analyses which directly demonstrates that the advantage of aQTLs in *trans* is not due to bias from inferring MRs by activity. Indeed, the use of expression-based MRs if anything gives slightly more advantage to *trans*-aQTLs versus eQTLs. Furthermore, Table 1 indicates that there are numerous GWAS loci that have *trans*-eQTLs with these MRs even in the absence of a corresponding *trans*-aQTL, which runs counter to the expectation that the stronger *trans*-aQTLs are due simply to higher correlation between activities and phenotype. The rationale for restricting *trans* analyses to MRs is that these putative MRs are much more likely to play a central role in the gene regulatory program instantiating the given phenotype. As such, it is most important to ensure our MR inference method is in fact picking out such important regulatory genes. Previous work from Andrea Califano’s lab has demonstrated that activity scores are more robust than expression values in this regard (Alvarez et al., 2016).

Reviewer #2: Hoskins et.al describe the application of aQTL analysis to identify alleles associated with cardiometabolic traits in fat tissue, including BMI, fat distribution, diabetes risk and blood cholesterol levels. While the global approach is not original, limiting the search to the genes/proteins most likely causally associated to the phenotype of interest dramatically boosts the statistical power of the GWAS/aQTL analysis and enrich the results in driver vs. passenger genes, allowing the discovery of potentially causal genetic-to-phenotypic associations that has not been previously described. This work constitutes an important contribution to the GWAS field. aQTL has the potential to transform GWAS analysis by providing regulatory-based mechanistic links between genomic and phenotypic traits and I expect to see many more reports using aQTL analysis.

Mayor points

1. Page #5, line 97: The authors state "Toward this end, we have developed a new method called activity QTL (aQTL) analysis that leverages tissue-specific gene expression regulatory networks to identify genetic effects on expression regulatory activities". However, the method they refer to is not new and have been previously described. See for example Chen et.al (2014). Identification of Causal Genetic Drivers of Human Disease through Systems-Level Analysis of Regulatory Networks. Cell, 159(2), 402–414. <https://doi.org/10.1016/j.cell.2014.09.021>. In fact, one of the steps of the DIGGIT algorithm, described in the reference above, is precisely aQTL analysis. This approach has been also used in other publications, such as Paull et.al. Cell 184:334 (2021), Bisikirska et.al. Cancer Res. 76:664 (2016), and Thorsson et.al. Immunity 48:812 (2018), among others. This previous work should be mentioned in the introduction and differences in the implementation with the author's method should be discussed.

We did not mean to imply that we were the first to test association between genotype and inferred activity. Rather, to our knowledge this is the first application of such an approach to elucidate the mechanisms mediating GWAS signals, and we have adjusted our language to better reflect this. We have included a brief statement contrasting our approach to the aQTL analyses in these listed studies in the last paragraph of the Introduction. As we understand it, DIGGIT, as applied to germline genetics, asks whether there are any upstream regulators of MRs that have any cis-eQTLs for variants very near their TSS, which the authors dub “F-SNPs”. Among those F-SNPs, DIGGIT then tests for aQTL associations with the relevant MRs. Consequently, DIGGIT is ideal for functional promoter variant identification from an MR-centric approach, rather than broad elucidation of the mediators of GWAS-identified genetic signals, which we are here attempting. For Paull et al., 2021, they first identified somatic mutations within upstream modulators of MRs, and then confirmed by aQTL that those mutations were indeed associated with MR activity. Thorsson et al., 2018 applied a similar analysis as Paull et al. Although Bisikirska et al., 2016 did report an MR analysis, we do not see any reporting of an aQTL-like analysis.

2. Page #8, line164: "Notably, while there is a high degree of correlation between these regulator's expression levels and respective activities, it is far from perfect, which highlights the important difference between these two metrics (Fig. S7C).". The authors need to be more specific. What does "high degree of correlation" means? What does "it is far from perfect" means? How the authors define "perfect" and "far"?

In addition, Figure S7C is impossible to interpret given the rows and columns have been clustered using different similarity inferences. This can be better displayed by clustering the genes based only on expression, only on inferred protein activity, or on an integration of both, but keeping the order of rows and columns the same, as to see the paired (gene-matched) correlation between expression and activity inferences in the diagonal of the heatmap. A different, maybe more effective way to show this data would be by plotting the distributions of the correlation coefficient for the gene-matched pairs and include in the same figure, as reference, the density distribution for the correlation coefficient for all the un-matched pairs. This should show that while there is strong correlation between expression and activity for some proteins, there will also be exceptions, specially for proteins whose activity is strongly post-translationally regulated.

We have cleaned up the wording and the figures for this as requested. The heatmap in Fig S7C is now clustered using the same similarity measures for columns and rows, putting the gene-matched correlations on the diagonal. Furthermore, we have added density plots of correlations between matched and unmatched expression and activities for MRs in Fig S7D and E.

3. Page #8, line 172: "...variants with genome-wide significant (i.e. P < 5E-8) associations...". Why this particular p-value threshold? Why not 1E-10 or 1E-5 for example? Multiple hypothesis correction?

5E-8 is the standard GWAS p-value threshold found ubiquitously throughout the GWAS literature to guard against false positives. It reflects a Bonferroni correction based on the number of independent (i.e., uncorrelated) SNPs (see PMIDs: 18300295, 18271029 or 18348202).

4. Please, consider referring to the MR proteins as "candidate MRs", since while their activity is statistically associated with the phenotype, they have not been experimentally validated as bona fide, causal drivers of the phenotype.

Throughout the manuscript we had inconsistently referred to MRs as “putative phenotypic MRs”. However, to better emphasize the unvalidated status of these MRs we have now more consistently referred to them as either “putative phenotypic MRs” or simply “candidate MRs”.

Minor points

1. Page #6, line 110: "Network edges in ARACNe co-expression networks are directed from regulator to target". This statement is not accurate. Since ARACNe edges are defined based on Mutual Information (MI), the edges are not directed. When the gene pair is constituted by a transcriptional regulator (TR) and non-TR protein, we can assume the direction is TR -> non-TR. However, when both proteins as TR or both are non-TR, the direction (i.e. which is the regulator and which is the target) is not clear.

This has been fixed. Thank you.

2. Page #7, line 143: "To further validate these phenotypic MRs for adipose, we used the METSIM expression..." This approach does not validate the candidate MRs as regulators of the phenotype, but test their association, and the RandomForest-model predictions, on an independent dataset. So I would suggest rephrasing to "To further test the candidate MRs on an independent dataset, we used the METSIM..."

This has been fixed. Thank you.

3. Page #8, line 161: The authors mention that the lack of clustering among MRs of the same phenotype suggest pleiotropy among MRs of these well correlated cardiometabolic phenotypes. However I do not think this is due too pleiotropy but simply because the cardiometabolic phenotypic traits represent only a minor component of the phenotypic (protein activity) variance of the dataset, and an unsupervised analysis as the one presented in Fig. S7B would capture only the major sources of phenotypic variance. The authors could corroborate this by performing a principal components analysis and checking the proportion of variance captures by the components showing significant association with the cardiometabolic phenotypes of their interest.

We agree with the reviewer and have removed our claim that this observation likely indicates pleiotropy.

4. Page #19, line 404: previously

This has been fixed. Thank you.

5. Consider adding to the discussion that by limiting the search for association to the candidate MRs of a particular phenotypic trait, aQTL analysis not only boosts the statistical power to find significant associations, but also does it for genes/proteins likely having a role as mechanistic regulators of the phenotype.

Yes, we were certainly trying to make this point, though perhaps too indirectly. We’ve made this more explicit now in the Discussion.

Reviewer #3: Review: Inferred expression regulator activities suggest genes mediating cardiometabolic genetic signals
Overview:

Hoskins et al. introduce a new method called activity QTL (aQTL) analysis to identify loci that have genetic effects on expression regulatory activities. This method is inspired by the issue that cis-eQTL only explains portion of GWAS mediating signals of complex traits and integrating trans-eQTL signals is underpowered due to large multiple testing burdens. As a result, authors hypothesize that GWAS functional variants have detectable impacts on the regulatory activities as reflected by X. Authors construct a regulatory network using transcriptomic data and expression regulators (TF, T co-F, etc.) and then inferred regulator activities scores. Using this network, the authors identify “master regulators” (MRs) for downstream trans-QTL analyses by prioritizing activities that predict cardiometabolic phenotypes. Then, authors performed cis-e/aQTL analyses on all expression regulator genes within 1MB of the significant GWAS SNPs and trans-e/aQTL analyses on MRs matched to the GWAS.

I find this area of research to be timely and important and the proposed approach interesting. The out-of-sample replication of random forests in METSIM data was particularly compelling. My comments are related mostly to providing additional overview/summary information of the main findings to help provide context for the interesting cases before discussing individual findings.

Major Comments:
1. In the abstract and introduction, it is unclear to me what quantity activity refers to specifically and how it is different from measured expression levels. Given, the central importance of this concept I think spending more space to providing appropriate context and definitions would be very helpful.

We have added two sentences to the abstract to summarize the general approach behind the calculation of activities and the identification of MRs.

2. The cis e/aQTL analysis in main text lacks summary information that would provide context to their findings. Namely, the total number of variant/gene pairs tested and the number of identified cis e/aQTLs identified at their given FDR level. (While table 1 alleviates this somewhat for trans e/aQTLs, a simple statement in the main text providing the total number of variant/gene pairs tested and the number of trans e/aQTLs identified would be helpful).

We thank the reviewer for his/her comments and have added a couple sentences in the Results section summarizing total variants, regulators and MRs tested in the various *cis* and *trans* analyses, as well as the total numbers of FDR significant *cis*-eRegulators, *cis*-aRegulators, *trans*-eMRs and *trans*-aMRs.

3. The authors note “two notable exceptions” where cis-aQTLs were stronger than correspond cis-eQTL effect sizes, however, it is not clear if two differences is expected or not, given the total number of tests performed (see above comment).

This is a good point. It would be easier to interpret the exceptionality of these cases with greater contextualization. We have therefore edited this section to be more explicit about the rarity of these exceptions and included a new supplemental table (Table S10) to summarize this comparison.

4. In comparing the proportions of identified QTLs with, the authors report a difference of 20% (cis) vs 72% (trans) of aQTLs having smaller p-values in compared to their eQTL counterparts. Can the authors attach a p-value to this statement using a proportion test or something similar? It isn’t clear what to expect based on the total number of identified cis vs trans QTLs.

An excellent idea! We have now run a Z-test of independent proportions for *cis* versus *trans* QTLs with more significant aQTLs, which yielded a *P* = 3.03E-280. This result has been added to the above referenced sentence.

5. In the colocalization analysis, it would be helpful to state directly (or in a table) the total number of risk regions examined, the total number of e/aQTLs that colocalized at some threshold (stratified by in cis vs in trans).

Thank you for the suggestion. More details on the full colocalization analysis have been added to the Results.

6. Recent work (Wang et al AJHG 2020; 10.1016/j.ajhg.2020.01.012) has demonstrated that GWAS signals are enriched for regions with increased enhancer complexity. It would be interesting to contrast enrichment of cis/trans aQTLs across respective EDS scores (see ref) compared with cis/trans eQTL. Namely, do colocalizing aQTLs do a better job characterizing risk regions with complex enhancer activity compared with eQTLs?

This was an excellent idea! We have now performed these analyses, which are detailed in a new Results subsection and a new figure (Fig 2) and supplemental table (Table S16).

Minor Comments:
1. Line 26 of the abstract: “outperformed” is slightly ambiguous. If it reflects identifying “explanatory genes”, I recommend being more explicit.

We have changed the wording to be less ambiguous.

2. While not critical, it would be interesting to perform an set enrichment or ontology analysis on the identified MR genes to provide context for putative function/pathways.

Yes, under most circumstances this would certainly be a good idea, and we did try it. However, given that our MR analyses are already restricted to expression regulator genes (i.e., transcription factors and cofactors and signal transduction factors) enrichments in gene ontologies and other gene sets have already been dramatically biased. Indeed, most of the top enriched gene sets among the MR lists are also among the top enriched gene sets when analyzing the full set of expression regulators. Therefore, rather than letting the enrichment rankings speak for themselves, we find ourselves sifting through uninformative gene sets to find relevant gene sets, which we worry invites bias into any conclusions.

Reviewer #4: Hoskins et.al., proposed an “activity QTL(aQTL) analysis” and hypothesized that genetic variants regulating disease-associated master regulators(MR) could underlie the GWAS signals for metabolic traits. The authors constructed gene co-expression network in forms of regulator-> targets connections at subcutaneous adipose, a tissue highly relevant to metabolic traits. They then inferred activities of regulators from the network. They next applied random forest regression to identify top regulators(MR) for each metabolic trait. With those information they performed analyses to identify cis/trans eQTLs/aQTLs that potentially regulate the expression or the activity of MRs in subcutaneous adipose and to test whether and how those QTLs colocalize with GWAS hits of metabolic traits. They found whilst in cis, regulatory variants that colocalize with GWAS hits seem to have a stronger effect for expression of MRs than for activity of MRs, in trans those variants tend to have a stronger effect for activity than for expression of MRs. They also provided examples where MR trans-QTLs(eQTLs or aQTLs) provided intriguing clues of the functional relevance of GWAS loci when cis-QTLs provided limited information. The authors concluded that these analyses can be broadly applied given the availability of data(expression and GWAS data) for many complex traits, and that expression/activity-type analyses of MRs add additionally valuable findings to classical global eQTL analyses.

Overall, I think this study proposed advanced and interesting concepts (aQTLs, MRs) and provided promising application (colocalization with GWAS signals). The analyses were carefully designed and the results are appropriately discussed. I have some questions which would like the authors to consider:

1. The author claimed that overall trans-aQTLs tend to be more significant/stronger than their respectively eQTLs. Was this conclusion drawn from raw p values from QTL analysis, or has the effect size been compared(if both activity and expression have been normalized onto comparable scale)? According to the paper, in its nature the activity of a MR reflected expression input from its target gene members within the regulon. Would it be possible that a strong tran-aQTL signal of a MR simply tracked a strong cis-eQTL signal of one of the target genes in the regulon of that MR?

Our assertion about e/aQTLs being stronger was based on *P*-values, though classification using effect size has a negligible difference in *cis* (only 25/27950, or 0.09% switch classification) and no difference in *trans*.

As for the hypothesis that a MR *trans*-aQTL could be driven by a large effect on a single target gene, we believe this is extremely unlikely. MR activities are calculated based on the expression of 216 target genes on average and the impact of each target gene on the inference is weighted by the confidence of the regulator-target interaction in the network and expression change matching the expected direction of effect (i.e., mode of regulation). Furthermore, the original VIPER paper (Alvarez et al., 2016) demonstrated that activity inferences remained robust even when up to 60% of regulon interactions were randomized or when 6 standard deviations of Gaussian noise was added to the expression data, which altogether suggest a change in just one target gene would have very little effect on the MRs inferred activity. Therefore, even a strong *cis*-eQTL for a target gene could not drive a false *trans*-aQTL effect unless that genetic signal also had *trans*-eQTL effects on many (maybe dozens) other downstream targets of the MR without that information flowing through their common upstream regulator, namely, the MR itself. We feel the likelihood of this is negligible.

2. A relevant and important question is that which variants, those interrupting/regulating MR or those interrupting/regulating MRs’ target genes in the regulon contribute more to the genetic susceptibility of complex traits? There has been debate in the field about the omnigenetic model(or the core-gene model) <https://www.ncbi.nlm.nih.gov/pmc/articles/PMC5536862/> and the polygenicity model <https://pubmed.ncbi.nlm.nih.gov/29906445/> for genetic architecture of complex traits. What the authors explored here are essentially in line with the ‘omnigenetic model’ by showing variants regulating core-genes(master regulators) with potentially larger effect can underlie some GWAS signals. What the author haven’t explored was that whether variants in target genes of the MRs also contributed to GWAS signals (polygenicity model), and how does that compare to the contribution from MR QTLs?. In figure s7, the author observed extensive correlation between MRs, both in terms of activity and expression. This suggested relatively limited independent pathways/room to be interrupted at core-genes(MRs) level and implicate peripheral genes(target genes of MRs) can potentially be better candidates for GWAS signals.

The reviewer raises an interesting question about how this work may fit into the debate about the “omnigenic” and “polygenic” models of genetic architecture of complex traits. Let us first point out that both models assume many variants are relevant to the instantiation of a complex trait or the development of a complex disease. Both sides also accept the importance of the complex regulatory networks through which information flows and affects the genes that are ultimately directly affecting the phenotype of interest. To our understanding, the main point of contention is whether one would expect stronger, more relevant variation in or around these ultimate effector genes (i.e. core genes) that would argue for an emphasis on rare variant analyses with WES to identify these “core genes”, rather than trying to catalog the vast amount of common variation scattered throughout the genome via GWAS and explore how such a complex array of genetic variation ultimately effects the complex trait. As such, we feel that our study does not provide much evidence supporting one view over the other, but instead attempts to empower the interpretation of how information for many common variants may flow through complex gene regulatory networks to ultimately impact the complex trait. This would likely be viewed as more useful to proponents of the “polygenic” model as they emphasize trying to understand the coordinated impact of the wealth of relevant, common variation identified by GWAS. Furthermore, we do not believe the MRs here studied meet the definition of a “core gene” as they are not the ultimate effectors of the phenotype and their individual activities are likely relevant to many phenotypes/cell states. Rather, we view their activities as a multidimensional reduction of their downstream impact on many genes, some of which may qualify as “core genes” under the “omnigenic” model. It is their coordinated activities that differentiate the various cell states associated with the phenotype of interest. However, given their prominent role in the cell state-specific gene regulatory program, we may expect the information from many of the GWAS variants scattered throughout the genome to flow through these MRs at some point. Also, we would like to be clear that we are not claiming these GWAS variants in *trans* are directly affecting the MRs expression or activity. In most cases we would expect the effect to be indirect.

As for investigating the impact of GWAS signals on *cis* target genes of MRs, this brings us right back to the general problems of relying on eQTLs to identify the genes that mediate the effects of GWAS signals, which was the motivation for this study. If we are curious about *trans* genetic effects on target genes of MRs, the question comes back to how that information flows from *trans* genetic signals to the target gene. Given the apparently important position of the MRs in the regulatory program and the connection between MR and target gene in the inferred tissue-specific regulatory network, it is reasonable to expect such *trans* effects to flow through the MRs themselves, which may be detectable by *trans*-eQTL/aQTL analyses when focused on such genes to reduce the multiple testing burden. Indeed, as we’ve reported, we are sometimes able to detect such effects on these MRs.

My other minor comments/questions are:
1. L150: whether the gender differences are responsible for the performance differences can be directly tested by only including females in test dataset. HDL and Triglycerides are potentially more fluctuated than other traits though.

The test TwinsUK data is already all female while the METSIM test set is all male. I’m afraid we cannot further stratify these test sets to get at the sex differences. This is a limitation to the study, and in response to Reviewer #1 we have better emphasized this limitation.

2. In some places the authors used ‘overlapped’ in other places they used ‘colocalized’. Are they interchangeable? Please be consistent.

We apologize if this was unclear, but they are not interchangeable. We were intentional in our usage of these terms. Overlapping refers to the presence of association signal for two traits at the same locus in the genome while colocalization is a formal statistical relationship between two genetic signals that accounts for association strengths and SNP LD structure to determine the likelihood that they share a common functional variant. This was mentioned in the Introduction, but we have now also expanded our description of the colocalization analysis in the Results section, which should hopefully make this distinction clearer.

3. The snp id is different in L214, legends of Figure 2 and the figure 2 itself. Why among the two independent SNPs, only one was considered?

When citing the *P*-values for the *KLF14* eQTL/aQTL signals in the Results section we had reported for rs4731702 as the top eSNP, but that SNP is highly correlated with rs972283 (r2 = 0.96) and therefore represents the same genetic signal. However, to avoid this confusion we have switched the text in the results to report the *P*-values for rs972283. As for why we focus on rs972283 more than rs287621 in the figures, though there is some suggestive evidence of at least two functional signals represented by these two SNPs, it would be an overstatement to claim they are independent. Their D’ = 1 while their r^2^ = 0.31 (due largely to differences in minor allele frequencies) in 1000 Genomes phase 3 European data. Consequently, there is still association observed between rs287621 and *KLF14* expression and activity, but judging by the colocalization analyses and LocusCompare plots, those associations seem due to rs287621’s correlation with rs972283 rather than because it represents a distinctly functional genetic signal relevant to *KLF14*. Of course, if it has a distinct functional relevance to T2D or triglycerides, then those effects must be mediated through some other gene, like perhaps *AC016831.7* or some gene in *trans*. This locus is very complicated, and consequently it is impossible to fairly capture all the relevant points in the space available in this manuscript. The figures provided are representative of the key points we are attempting to convey. However, all the eQTL/aQTL and colocalization results are included in the Supplemental Tables if one were inclined to dig deeper into the matter.
