# Supplementary material for: Inferred expression regulator activities suggest genes mediating cardiometabolic genetic signals
Source: PLoS Comput Biol. 2021 Nov 18;17(11):e1009563. doi: 10.1371/journal.pcbi.1009563 (PMC8639061; doi:10.1371/journal.pcbi.1009563)
Supplement: Attachment — Submitted filename: review.docx [file pcbi.1009563.s035.docx]

The author has addressed my questions.
